# Dermatologic Conundrum: A Cardiac Condition Masqueraded as a Dermatologic Distraction

**DOI:** 10.1155/2020/5314503

**Published:** 2020-03-11

**Authors:** George Degheim, Evan Hiner, Abeer Berry, Nathan Foster

**Affiliations:** Providence Hospital and Medical Centers Michigan State University South Campus, Southfield, MI, 48075, USA

## Abstract

A 38-year-old male presented to the emergency department (ED) complaining of extreme pain and a petechial rash on the left ankle for two weeks associated with generalized fatigue, intermittent fevers, and weight loss. He was discharged home from the ED on pain medications. He returned a few days later with a progressive rash that involved the entire left lower extremity to the level of the knee. He was diagnosed with herpes zoster (shingles) and was prescribed acyclovir and steroids. After several days, the patient presented for the third time to the ED. He developed a right lower extremity discomfort this time. The pain in bilateral lower extremities had become unbearable. His cardiac examination revealed a systolic murmur at the apex and a faint diastolic murmur at the left sternal border. Ultimately, he had an echocardiogram that demonstrated both a bicuspid aortic valve and large vegetation on the anterior leaflet of the mitral valve, and his blood culture grew *Streptococcus mitis* and *Streptococcus oralis*. The patient was subsequently diagnosed with subacute bacterial endocarditis thought to be sourced from his poor dentition. The diagnosis of infective endocarditis is often delayed due to its nonspecific clinical presentations. Our case displays an unusual skin manifestation of IE that may be present in the absence of other signs and symptoms of the disease.

## 1. Introduction

The epidemiology of infective endocarditis (IE) has significantly changed in the past decade due to healthcare-associated IE, advances in structural heart disease, and valvular replacements. In the United States, the incidence of IE is estimated to be near 15,000 cases per year [1, 2]. IE may present with a broad spectrum of clinical symptoms and complications influenced by several causes. These include the virulence of the infecting organism, degree of local tissue damage of the involved cardiac valves, embolization to both the systemic and pulmonary circulation, and consequence of circulating immune complexes [1]. A number of associated conditions have been shown to increase the risk of IE. These include chronic intravenous drug abuse, prior history of IE, the presence of intravenous access for medical care, prosthetic devices, and implantable cardiac devices [1].

IE is a well-known clinical entity. However, despite advances in diagnostic techniques, it continues to be clinically challenging which results in significant morbidity and mortality. The in-hospital mortality rate remains 15–20% with a five-year mortality approaching 40% [2]. IE typically presents with clinical manifestations involving multiple organ systems. Our case focuses two important concepts: (1) patients with bicuspid aortic valves (BAV) remain a potential high-risk group for infective endocarditis and (2) the dermatologic findings of IE that demonstrate the necessity of a thorough skin examination when a high index of suspicion for IE is present.

## 2. Case Report

We present a 38-year-old Caucasian male who was brought to the emergency department (ED) complaining of extreme pain and an erythematous petechial crusted rash on his lower extremity (ankle) for two weeks. In addition, he suffered from one month of generalized fatigue. Associated symptoms included drenching night sweats, intermittent fevers, and weight loss of 10 pounds. He denied any history of intravenous drug abuse, congenital heart disease, or prior cardiac surgeries. There was no mention of an auscultated murmur on exam. No clear diagnosis was entertained at that time. He was discharged home from the ED with support care and follow-up with primary care.

He returned to the ED a few days later with similar complaints of left lower extremity pain. His rash had progressed and now involved his entire left lower extremity to the level of the knee. Due to the presence of pain and crusted skin lesions, a clinical diagnosis of herpes zoster (shingles) was entertained. The patient was prescribed acyclovir and steroids for one-week duration. Despite this regimen, his symptoms and lower extremity rash did not resolve. The intermittent fevers and night sweats intensified and became more frequent.

**Figure 1:**
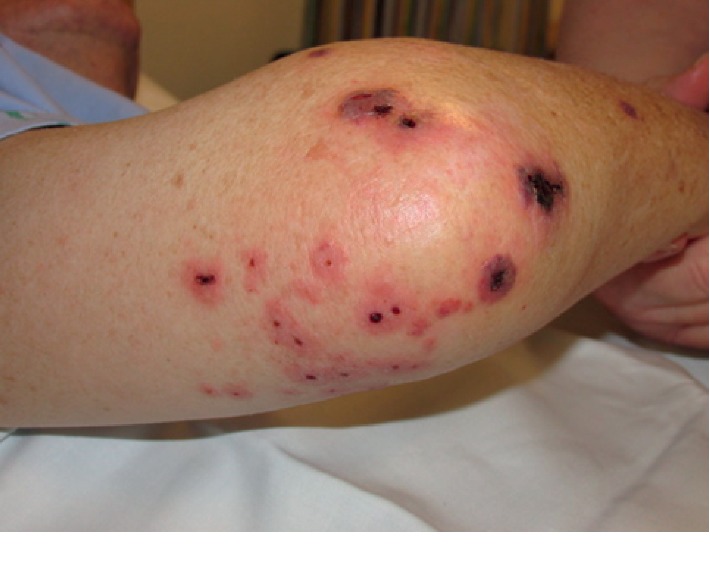
Showing the petechial rash on the anterior aspect of the lower extremity.

After several days, right lower extremity discomfort developed and made it difficult to ambulate. The pain in bilateral lower extremities had become unbearable. The patient then returned to the emergency department for the third time. His presenting vital signs revealed a temperature of 99.5 degrees Fahrenheit. The remaining vital signs were within normal limits. He was awake, alert, and oriented but appeared anxious and diaphoretic. There was an erythematous petechial rash with crust present on the anterior aspect of his left lower extremity (Figure 1). His cardiac examination revealed a 3/6 systolic murmur best heard at the apex and a 2/6 diastolic murmur at the left sternal border. His lower extremities were tender to minimal palpation. His left dorsalis pedis pulse was diminished in comparison with the right side. There were no other pertinent exam findings.

**Figure 2:**
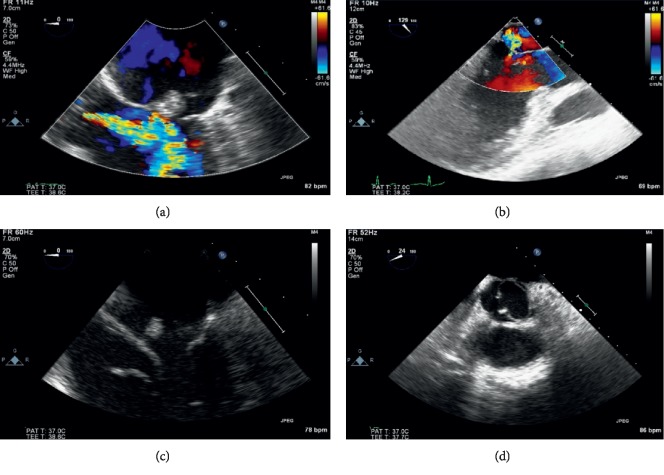
This demonstrates a transesophageal echocardiogram for evaluation of endocarditis. (a): Mid esophageal view showing a large vegetation on the anterior leaflet of the mitral valve. The color doppler shows severe aortic regurgitation jet towards the anterior mitral leaftet. (b): Mid esophageal long axis view demonstrating severe mitral regurgitation. (c): Mobile echo density on the atria aspect of the mitral valve. (d): Aortic valve is found to be a bicuspid valve associated with severe aortic regurgitation.

His laboratory findings revealed no significant leukocytosis but an elevated erythrocyte sedimentation rate and C-reactive protein level. Ultimately, a transesophageal echocardiogram performed showed normal left ventricular systolic function and a large vegetation on the anterior leaflet of the mitral valve with associated severe aortic and mitral regurgitation (Figure 2). The aortic valve was also noted to be bicuspid. His blood cultures isolated two *Streptococcus* species: *Streptococcus mitis* and *Streptococcus oralis*.

The patient was subsequently diagnosed with subacute bacterial endocarditis thought to be sourced from his poor dentition. The rash was likely due to septic emboli with immune complex deposition resulting in a leukocytoclastic vasculitis. He was treated with antibiotics and underwent both mitral and aortic valve replacements. Postoperatively, he required pacing due to the development of complete heart block. A dual-chamber permanent pacemaker was subsequently placed. The patient's symptoms improved significantly, and he was eventually discharged home in a stable condition.

## 3. Discussion

Infective endocarditis (IE) is a life-threatening disease that leads to significant morbidity and mortality. Despite advances in its diagnosis and treatment, the recognition is often delayed due to its nonspecific clinical presentations. The diagnosis of IE can be established with highly sensitive (80%), specific (99%) diagnostic criteria, the Duke criteria, which have been developed to reflect the clinical, laboratory, and echocardiographic findings of IE [3, 4].

The frequency of IE cases that present with cutaneous lesions alone varies from 5% to 25% with an average of 11.9% [5]. The most common dermatologic presentation among these cases is purpura. According to the literature study, purpura is most commonly distributed on the lower aspects of the body and infrequently identified on mucosal surfaces [5]. The underlying mechanism for the skin manifestation is immune complex deposition. This is primary caused by septic emboli that result in an inflammatory cascade-releasing cytokines which can stimulate cytokine receptors often resulting symptoms [6]. This skin phenomenon was identified by Sir William Osler in the 20th century. Additional skin manifestations included in Duke's criteria are Osler's nodes and Janeway lesions. Osler's nodes are described as being painful, erythematous, raised lesions found on the distal extremities. Janeway lesions are nontender, erythematous, or hemorrhagic macular or nodular lesions of the palms or soles [7]. It was found that patients who presented initially with skin manifestations of infective endocarditis had a higher rate of extracardiac complications than patients without skin manifestations (32.8% vs. 18.4%, respectively) [5, 8]. Important to our case is that patients with bicuspid aortic valves (BAV) were found to have a higher risk for skin manifestation [5]. The skin manifestations may correlate with the severity of IE since they represent an underlying embolic process. Various studies demonstrate that up to 20% of the cases of IE might result from a cutaneous entry [5]. Therefore, a systematic and comprehensive dermatological examination is critical for the rapid recognition and management of IE [5].

A number of associated conditions have been shown to increase the risk of IE. These include intravenous drug abuse, prior history of IE, the presence of long-term intravenous access for medical care, prosthetic valves, and cardiac implantable electronic devices. The above conditions mentioned have become the high-risk groups requiring endocarditis prophylaxis. In 2007, the American Heart Association (AHA) removed two prior cardiac conditions for endocarditis prophylaxis [8]. Patients with mitral valve prolapse and bicuspid aortic valve (BAV) are now restricted from prophylactic antibiotic use; however, recent data show an increased incidence of infective endocarditis within this population with an increased risk of perivalvular extension with abscess formation [9, 10]. Based on congenital valvular literature, BAV is the most common congenital heart disease with estimated prevalence of around 0.5–2.0% [10]. According to recent literature studies, the incidence of IE is about 236 cases per 100,000 individuals per year and represents nearly a 30-fold higher risk of IE compared to the general population [10]. Interestingly, the population at highest risk includes patients at a younger age and males, both which are represented in our case. This case emphasizes the important concept that patients with BAV are at higher risk for cardiovascular complications including aortic valve disease, infective endocarditis, and other manifestations not shown in our case.

Our case displays the skin manifestations secondary due to the immunologic phenomena of IE that may be present in the absence of other signs and symptoms of the disease. Physicians need to investigate further to rule out IE in these scenarios especially since IE-associated skin findings can indicate a greater severity of illness and worse outcomes. Additionally, bicuspid aortic valve remains the most common congenital heart disease, often silent and unknown to the patient, and individuals need to be considered at a higher risk for IE.

## 4. Conclusion

Our case illustrates atypical signs and symptoms of IE that complicated the initial diagnosis. This delay is not uncommon and leads to increased morbidity and mortality associated with IE. It is important to be cognizant of the skin manifestations of IE as they may represent increased infection burden and severity. Skin findings are typically associated with embolic events and may foreshadow the development of worse sequelae. A systematic evaluation by an experienced physician is essential to establish an early diagnosis. Our findings stress the necessity of a complete physical examination to aid with determining the diagnosis rather than relying solely on diagnostic studies.

## Additional Points

Learning objectives: it is important to recognize the skin manifestations of infective endocarditis as they may be the initial presenting symptoms and often represent an increased infection burden and severity. Patients who presented initially with skin manifestations are found to have a higher rate of extracardiac complications compared with patients without skin manifestations. Skin findings are typically associated with embolic events and may foreshadow the need for urgent cardiovascular surgery associated with worsening morbidity and mortality.
